# Speech Characteristics of Childhood Apraxia of Speech in Hebrew-Speaking Children

**DOI:** 10.3390/children13091155

**Published:** 2026-08-28

**Authors:** Natalie Watter, Edwin Maas, Osnat Segal

**Affiliations:** 1 Department of Communication Disorders, Faculty of Medicine, Tel Aviv University, Tel Aviv 6997801, Israel; watter@mail.tau.ac.il; 2 Department of Communication Sciences and Disorders, Temple University, Philadelphia, PA 19122, USA; emaas@temple.edu; 3 Department of Communication Disorders, Gray Faculty of Medical and Health Sciences, Sagol School of Neuroscience, Tel Aviv University, Tel Aviv 6997801, Israel

**Keywords:** childhood apraxia of speech, Hebrew, speech characteristics, diagnosis, lexical stress, speech–language pathologists

## Abstract

**Highlights:**

**What are the main findings?**

- Israeli speech–language pathologists identified inconsistent errors, difficulty with multisyllabic words, and syllable sequencing difficulties as the most common and diagnostically informative characteristics of childhood apraxia of speech.
- Prosodic features, particularly lexical stress errors, were perceived as relatively uncommon and less diagnostically informative in Hebrew-speaking children with childhood apraxia of speech.

**What are the implications of the main findings?**

- The findings suggest that some clinical manifestations of childhood apraxia of speech may be influenced by language-specific phonological and prosodic properties.
- Cross-linguistic evidence should inform the development of language-appropriate diagnostic frameworks and assessment tools for childhood apraxia of speech.

**Abstract:**

**Purpose:** Childhood apraxia of speech (CAS) is a motor speech disorder characterized by deficits in speech planning and programming. However, current diagnostic frameworks have been developed primarily from studies of English-speaking children, raising questions regarding their applicability to other languages. This study examined the common and diagnostic speech characteristics of Hebrew-speaking children with CAS as perceived by Israeli speech–language pathologists (SLPs). **Method:** Two hundred Israeli SLPs with experience diagnosing and/or treating children with CAS completed an online questionnaire. Participants rated 26 speech characteristics on 7-point scales reflecting their perceived frequency and diagnostic specificity in Hebrew-speaking children with CAS. Descriptive analyses included mean ratings and the percentage of clinicians endorsing each characteristic. **Results:** Increased difficulty with multisyllabic words (*M* = 6.37) and inconsistent errors (*M* = 6.32) received the highest frequency ratings and were endorsed by 87.0% and 84.0% of clinicians, respectively, as common characteristics of CAS. Inconsistent errors received the highest diagnostic specificity rating (*M* = 6.12), followed by groping movements (*M* = 5.73) and difficulty sequencing syllables (*M* = 5.48). Prosodic features, including lexical stress errors (*M* = 3.72) and equal stress patterns (*M* = 3.73), received ratings at or below the midpoint of the scale and were endorsed by fewer than 20% of clinicians as common characteristics of CAS. Breathy voice (*M* = 3.11) and consistent hypernasality (*M* = 2.79), two control characteristics not typically associated with CAS, received the lowest ratings. **Conclusions:** Israeli SLPs identified syllable sequencing difficulties and inconsistency as the most salient and diagnostically informative characteristics of CAS in Hebrew-speaking children. In contrast, prosodic features, particularly lexical stress errors, were perceived as less common and less central to diagnosis than suggested by English-based diagnostic frameworks. These findings suggest that the clinical manifestation of CAS may be influenced by language-specific phonological and prosodic properties and underscore the need for direct investigation of CAS characteristics in Hebrew-speaking children.

## 1. Introduction

Childhood apraxia of speech (CAS) is a rare but clinically significant pediatric speech sound disorder characterized by impairments in the planning and programming of speech movements, resulting in reduced precision and consistency of speech production in the absence of neuromuscular deficits [1]. Children with CAS often demonstrate inconsistent speech errors, disrupted coarticulatory transitions, and prosodic abnormalities, particularly in lexical stress production [1,2]. These difficulties can significantly affect speech intelligibility, language development, literacy acquisition, academic achievement, and psychosocial functioning throughout childhood and adolescence [3,4,5]. Although research on CAS has increased substantially over the past two decades, current understanding of its clinical characteristics, diagnostic markers, and intervention approaches is based primarily on studies of English-speaking children [6,7,8,9]. Consequently, the applicability of existing diagnostic frameworks to children who speak other languages remains uncertain.

Increasing cross-linguistic evidence suggests that the clinical manifestation of CAS may differ across languages, reflecting language-specific phonological, prosodic, and phonotactic properties [10,11,12,13,14]. For example, inconsistent errors and sequencing difficulties appear to be common across languages, including Persian, Swedish, and Cantonese [10,12,13]. In contrast, prosodic abnormalities such as lexical stress errors and equal stress patterns, which are commonly reported features in English-speaking children with CAS [1,2,15], appear to be less prominent or less diagnostically useful in some other languages [10,12,13,14]. Similarly, language-specific features such as intrusive schwa insertion may occur in English but not in French-speaking children with CAS because of differences in phonological structure and syllable organization [11]. Together, these findings suggest that CAS may not manifest identically across languages and that speech characteristics should be interpreted within the linguistic context of the child’s native language. Table 1 summarizes the principal cross-linguistic survey studies examining speech–language pathologists’ perceptions of the speech characteristics of CAS.

Hebrew provides a particularly important context for investigating the speech characteristics of CAS due to its unique phonological and prosodic structure. Unlike English, Hebrew predominantly exhibits word-final stress and lacks vowel reduction in unstressed syllables [16,17]. These characteristics may influence the manifestation of prosodic impairments in children with CAS. Consistent with this possibility, recent evidence demonstrated that Hebrew-speaking children with CAS produced relatively accurate lexical stress patterns compared to English-speaking children with CAS [18]. In addition, Hebrew has a relatively small vowel inventory consisting of five vowels, which may reduce the perceptual salience of vowel distortions compared to languages with larger vowel systems, such as English [17]. Such linguistic differences suggest that some speech characteristics commonly associated with CAS in English may present differently in Hebrew-speaking children.

Research on CAS in Hebrew-speaking children remains extremely limited. Existing studies have primarily focused on lexical stress production [18], while little is known about the broader speech characteristics of Hebrew-speaking children with CAS, including segmental errors, sequencing difficulties, speech inconsistency, and motor speech behaviors [19]. Furthermore, no consensus currently exists regarding which speech characteristics are most frequent or most diagnostically informative in Hebrew. This gap is clinically significant because speech–language pathologists rely heavily on perceptual speech characteristics when diagnosing CAS, particularly in the absence of objective biomarkers or universally accepted diagnostic tests [2,6].

Consequently, understanding how CAS manifests in Hebrew is essential for improving diagnostic accuracy and developing language-appropriate clinical frameworks. Although frameworks such as the ASHA (2007) consensus criteria and Strand’s Mayo Clinic checklist [1,20,21] have contributed substantially to the field, these criteria were largely developed based on English-speaking populations. Thus, it remains unclear whether the same speech characteristics are equally relevant and observable in children who speak typologically different languages, such as Hebrew. Examining CAS across languages may therefore help distinguish universal motor speech characteristics from language-specific manifestations shaped by linguistic structure.

Therefore, the purpose of the present study was to examine the speech characteristics of Hebrew-speaking children with CAS from the perspectives of Israeli speech–language pathologists (SLPs). Specifically, the study sought to address two research questions: (1) Which speech characteristics do Israeli SLPs perceive as most common in Hebrew-speaking children with CAS? (2) Which speech characteristics do they perceive as most diagnostically indicative of CAS?

By documenting clinicians’ observations and diagnostic judgments, this study extends previous survey research conducted in English, Persian, Swedish, Cantonese, Estonian, Finnish, and Lithuanian to Hebrew. More broadly, the findings may contribute to distinguishing universal characteristics of CAS from language-specific manifestations shaped by linguistic structure, thereby informing cross-linguistic models of CAS assessment and diagnosis.

## 2. Method

### 2.1. Participants

The study included 200 SLPs who met the following inclusion criteria: Each SLP (a) was a qualified and licensed SLP, (b) completed speech–language pathology training in Israel, (c) currently practiced in Israel and treated Hebrew-speaking children, and (d) had current or previous experience diagnosing and/or treating children with childhood apraxia of speech (CAS). Demographic characteristics of the participants are presented in Table 2.

Nearly all participants were women (98.5%) and native Hebrew speakers (95.5%). Participants represented a broad range of ages, educational backgrounds, and years of clinical experience. Most participants were between 30 and 39 years of age (53.5%), held either a bachelor’s or master’s degree, and had between 5 and 10 years of professional experience. All participants reported Hebrew as their primary language of clinical practice, although some also provided services in additional languages, including English, Russian, French, and Yiddish.

Participants represented a variety of clinical work settings (Table 3).

The most common workplaces included private speech and language clinics, special preschool and primary schools for children with speech and language disorders, and child development centers. Most participants reported working across one or two clinical settings.

**Figure 1:**
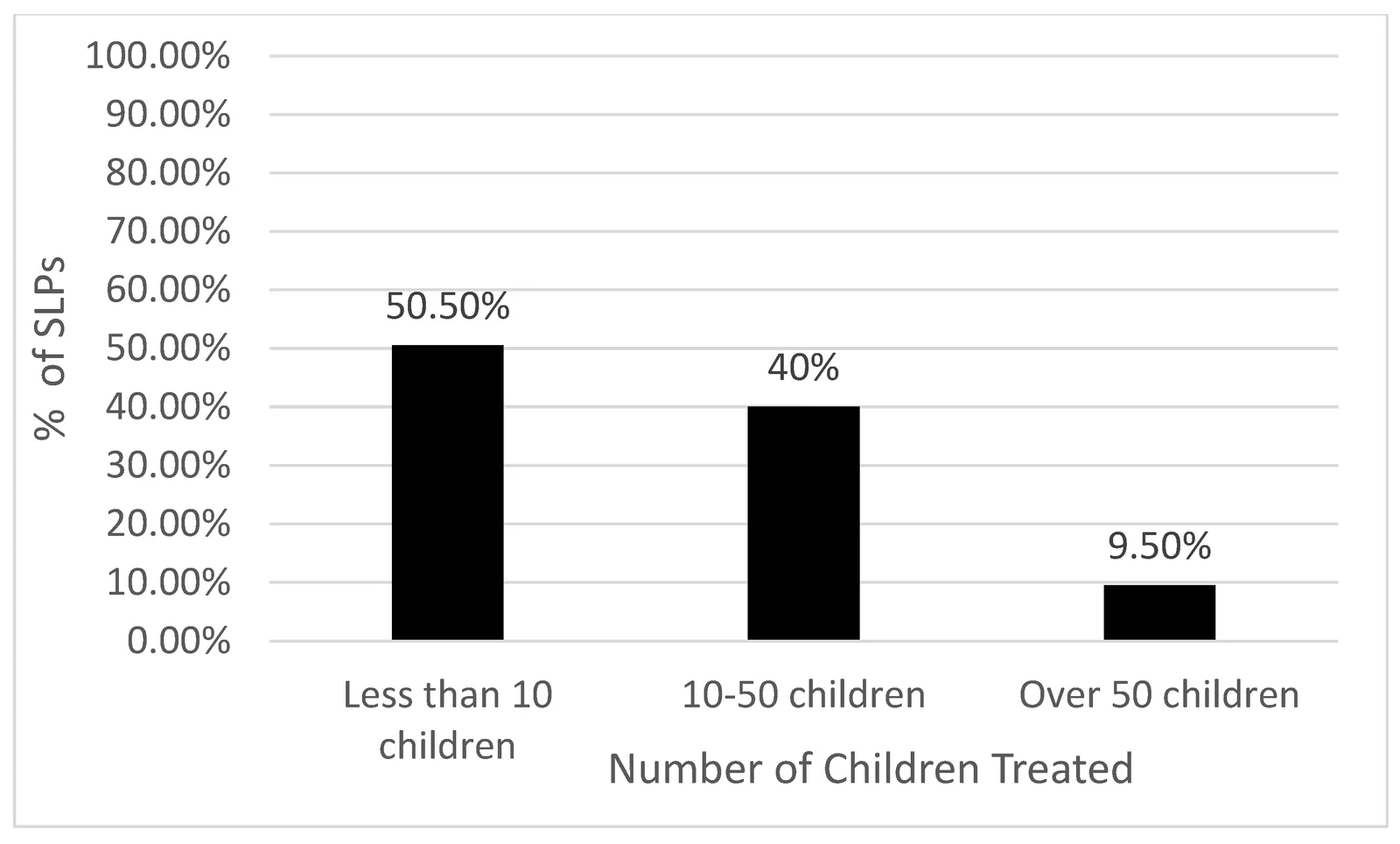
Number of children with CAS treated by SLPs.

Most participants (91.5%) reported current clinical experience working with children with CAS. Participants varied in the number of children with CAS they had treated, ranging from fewer than 10 children to more than 50 children across their clinical careers (Figure 1). The primary age range of children treated was 3–6 years.

### 2.2. Materials

A questionnaire was developed to examine SLPs’ perceptions of the common and diagnostic characteristics of Hebrew-speaking children with CAS. The questionnaire was based on previous surveys [20,21] and clinical checklists [1,8,9]. It included the following three sections: (a) demographic information (e.g., education, workplace, years of experience); (b) experience with CAS, including clinical exposure and sources of knowledge; and (c) clinical features of CAS.

The section on clinical features of CAS included 26 items assessing both the common speech characteristics associated with CAS and the characteristics considered diagnostically indicative of the disorder. Items were selected to represent speech characteristics consistently described in the CAS literature across word-level sequencing (e.g., syllable deletions), segmental (e.g., consonant substitutions), prosodic (e.g., lexical stress errors), and non-verbal motor speech domains (e.g., groping movements). Two unrelated “false” characteristics (consistent hypernasality and breathy voice) were also included to control for response bias. The wording of the items was adapted for use with Hebrew-speaking clinicians while preserving the terminology used in previous surveys and clinical frameworks. The questionnaire was translated into Hebrew and independently back-translated into English to ensure conceptual equivalence. Prosodic items referred to lexical stress errors, phrasal stress errors, and atypical intonation. To ensure consistent interpretation in Hebrew, lexical stress errors were defined as incorrect placement of word stress, phrasal stress errors as inappropriate stress assignment within phrases, and atypical intonation as deviations in sentence-level intonation patterns (Appendix A). The questionnaire was reviewed by the study authors and two additional Israeli experts in childhood apraxia of speech, and the wording of several items was refined based on their feedback to improve clarity and content validity. A pilot version of the questionnaire was subsequently administered to five speech–language pathologists to evaluate the clarity and comprehensibility of the items, and minor wording revisions were made prior to distribution.

For frequency ratings, 1 indicated “not common at all” and 7 indicated “very common” in children with CAS. For diagnostic value ratings, 1 indicated “not specific/indicative at all”, and 7 indicated “very specific/indicative” of CAS diagnosis.

Internal consistency was high for the clinical features ratings, with Cronbach’s α values of 0.87 for frequency ratings and 0.84 for diagnostic value ratings [22]. The full questionnaire appears in Appendix A.

### 2.3. Procedure

Ethical approval for this study was obtained from the Ethics Committee of Tel-Aviv University (0008964-1). All participants provided informed consent before accessing the questionnaire. Participation was voluntary, and responses were kept confidential. To maintain anonymity, the questionnaire was administered electronically through Qualtrics (Qualtrics, Provo, UT, USA; web-based platform, version not applicable) using an anonymous link that did not collect identifying information, such as email addresses or other personal identifiers.

The questionnaire was distributed through multiple channels, including professional Facebook and WhatsApp groups, professional mailing lists, and the Israeli Speech, Hearing, and Language Association (ISHLA). Data were collected over a four-month period. A total of 253 SLPs initiated the survey. Eligibility screening questions were incorporated to ensure that participants met all inclusion criteria, including current or previous experience diagnosing and/or treating children with childhood apraxia of speech (CAS). Respondents who did not meet the eligibility criteria (*n* = 32) and incomplete surveys with less than 95% completion (*n* = 21) were excluded from analysis, yielding a final sample of 200 SLPs. This screening procedure was intentionally designed to ensure that responses reflected the perspectives of clinicians with relevant experience working with children with CAS. Participants were entered into a raffle for three 500 New Israeli Shekel (NIS) gift cards.

Participants first completed demographic questions and provided information regarding their clinical experience with CAS. They then rated the frequency of speech characteristics associated with CAS, followed by the extent to which they considered each characteristic diagnostically indicative of the disorder. Surveys were considered valid if respondents met all inclusion criteria and completed at least 95% of the questionnaire items.

### 2.4. Statistical Analysis

Data were analyzed using IBM SPSS Statistics for Windows, Version 27.0 (IBM Corp., Armonk, NY, USA). Descriptive statistics were calculated for all study variables. Frequencies and percentages were used to summarize participants’ demographic and professional characteristics. For each speech characteristic, mean ratings and standard deviations were calculated separately for perceived frequency and diagnostic specificity. In addition, the percentage of SLPs assigning ratings of 6 or 7 on the 7-point scales was calculated to identify characteristics perceived as most common and diagnostically specific to CAS. Speech characteristics were ranked according to their mean frequency and diagnostic specificity ratings.

## 3. Results

### 3.1. Perceived Common CAS Characteristics in Hebrew-Speaking Children

**Figure 2:**
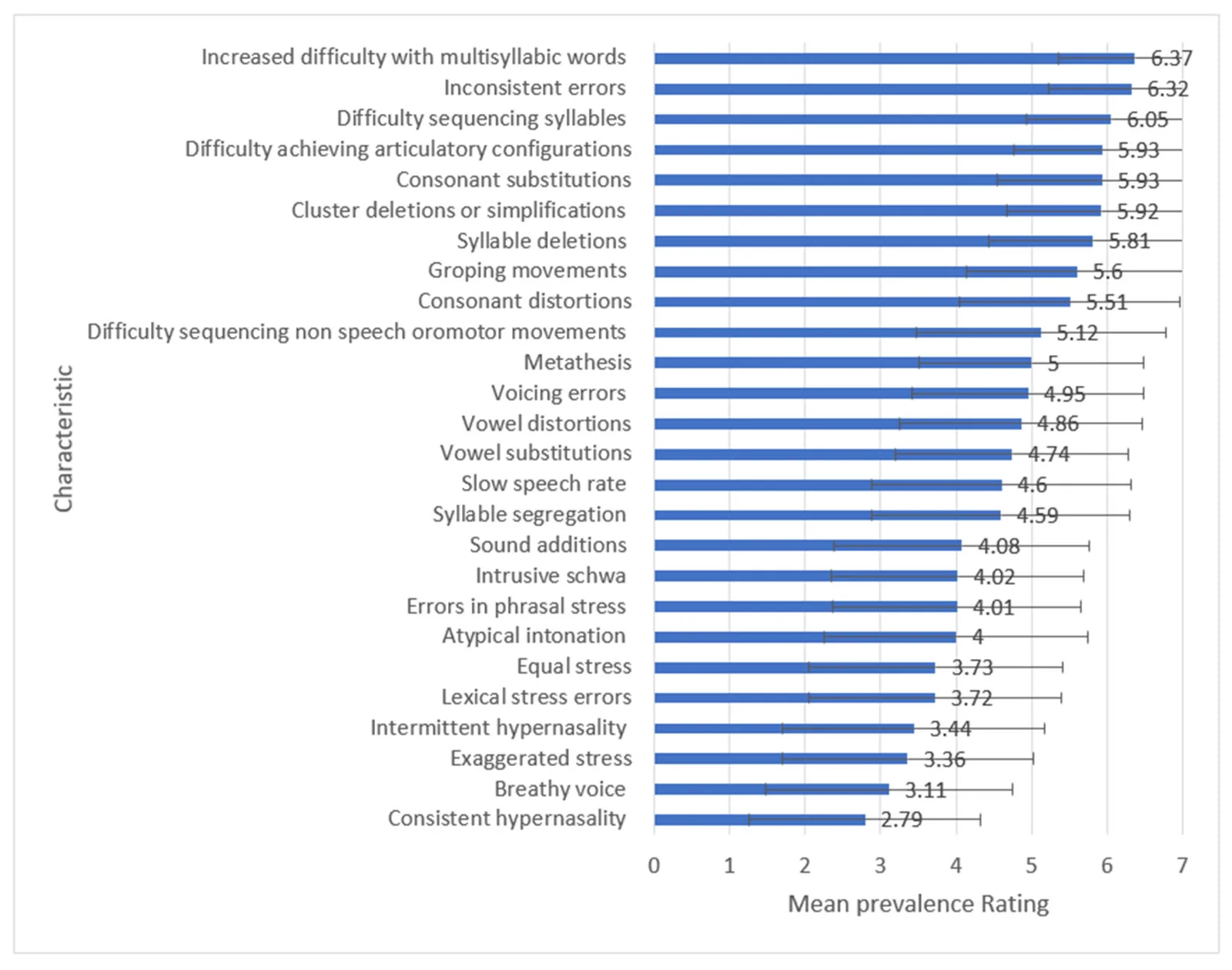
Mean frequency ratings of speech characteristics in Hebrew-speaking children with CAS as reported by SLPs. Scale: 1–7, where 1 = not common at all and 7 = very common. Error bars represent ±1 standard deviation. Breathy voice and consistent hypernasality were included as control characteristics and are not considered core features of CAS.

Figure 2 presents the mean frequency ratings for each speech characteristic on a 7-point scale, where 1 indicated “not common at all”, and 7 indicated “very common”.

Clinicians consistently identified increased difficulty with multisyllabic words, inconsistent errors, and difficulty sequencing syllables as the most common characteristics of CAS. Several additional speech characteristics, including difficulties achieving articulatory configurations, consonant substitutions, cluster deletions or simplifications, groping movements, and difficulty sequencing non-speech oromotor movements, were also perceived as common. In contrast, prosodic characteristics generally received lower frequency ratings, with mean ratings at or slightly below the midpoint of the 7-point scale. The two control characteristics, breathy voice and consistent hypernasality, received the lowest ratings (Figure 2).

**Figure 3:**
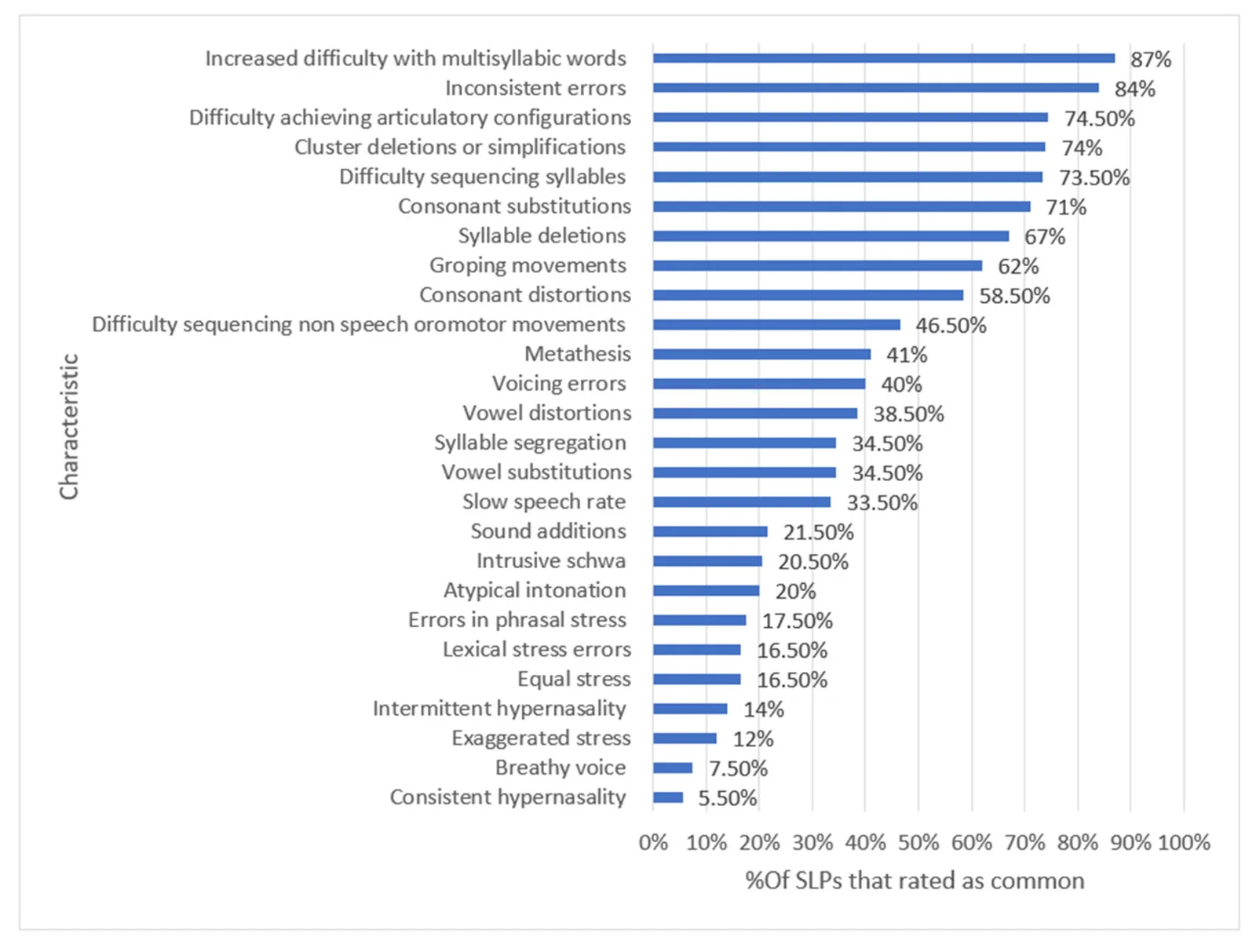
Percentage of SLPs rating each CAS characteristic as common (ratings of 6–7 on the 7-point scale). Higher percentages indicate greater clinical agreement regarding the common occurrence of the characteristic in children with CAS. Note: Breathy voice and consistent hypernasality were included as control characteristics and are not considered core features of CAS.

Figure 3 presents the percentage of SLPs who rated each characteristic as common (ratings of 6–7 on the 7-point scale).

There was strong agreement among clinicians regarding the characteristics most commonly associated with CAS. Increased difficulty with multisyllabic words and inconsistent errors received the highest levels of endorsement, followed by difficulties achieving articulatory configurations, cluster deletions or simplifications, difficulty sequencing syllables, and consonant substitutions. In contrast, prosodic characteristics were endorsed as common by fewer than one-fifth of clinicians, and the two control characteristics, breathy voice and consistent hypernasality, received the lowest levels of endorsement (Figure 3).

### 3.2. Perceived Diagnostic CAS Characteristics in Hebrew-Speaking Children

**Figure 4:**
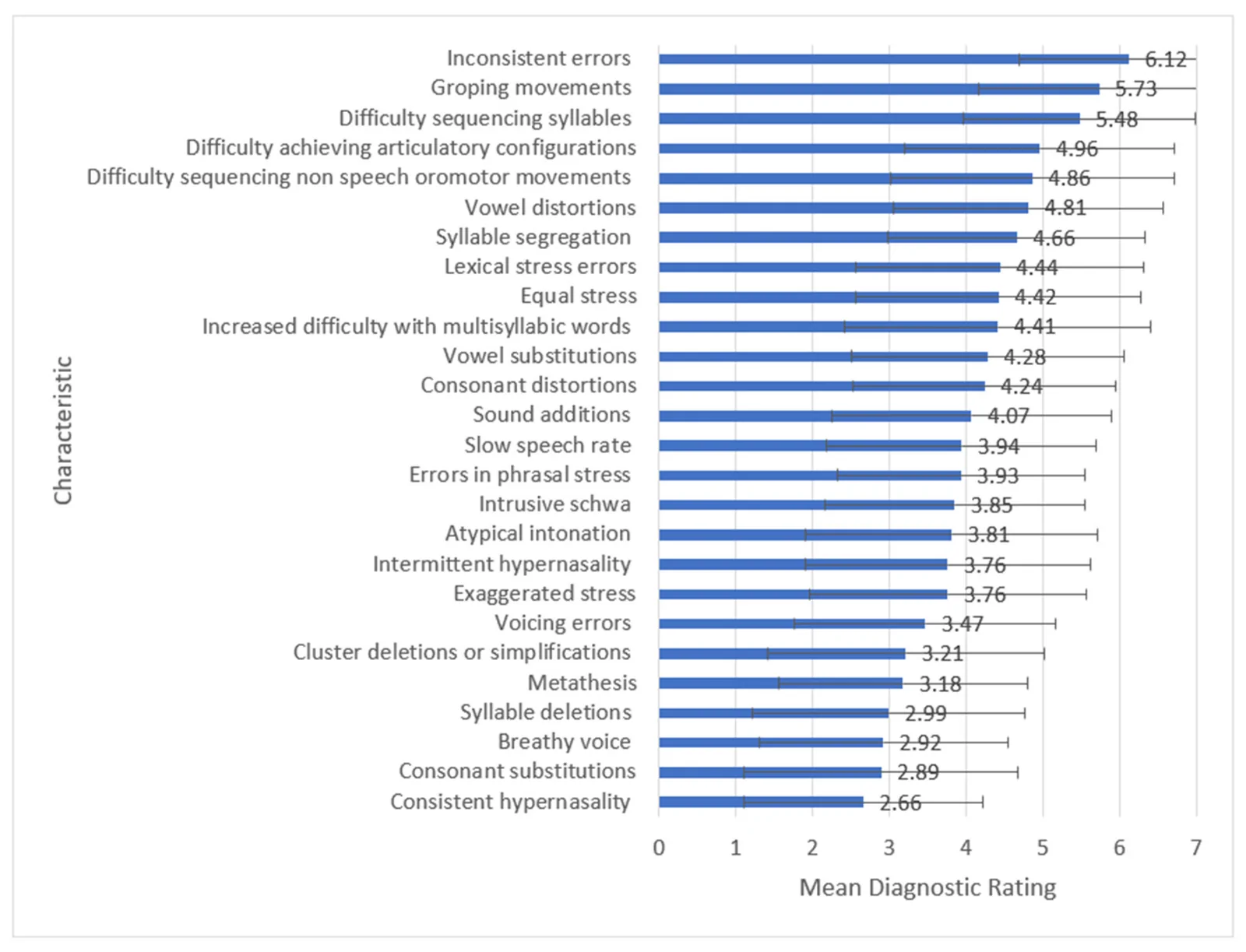
Mean diagnostic ratings of CAS characteristics as reported by SLPs. Scale: 1–7, where 1 = not common at all and 7 = very common. Error bars represent ±1 standard deviation. Breathy voice and consistent hypernasality were included as control characteristics and are not considered core features of CAS.

Figure 4 presents the mean diagnostic specificity ratings for each speech characteristic on a 7-point scale, where 1 indicated “not diagnostically specific at all”, and 7 indicated “highly diagnostically specific.”

Inconsistent errors were perceived as the most diagnostically specific characteristic of CAS, followed by groping movements and difficulty sequencing syllables. Difficulty achieving articulatory configurations, difficulty sequencing non-speech oromotor movements, and vowel distortions were also considered relatively diagnostic. Lexical stress errors, equal stress patterns, and increased difficulty with multisyllabic words received ratings slightly above the midpoint of the 7-point scale. In contrast, the two control characteristics, breathy voice and consistent hypernasality, received the lowest diagnostic specificity ratings (Figure 4).

**Figure 5:**
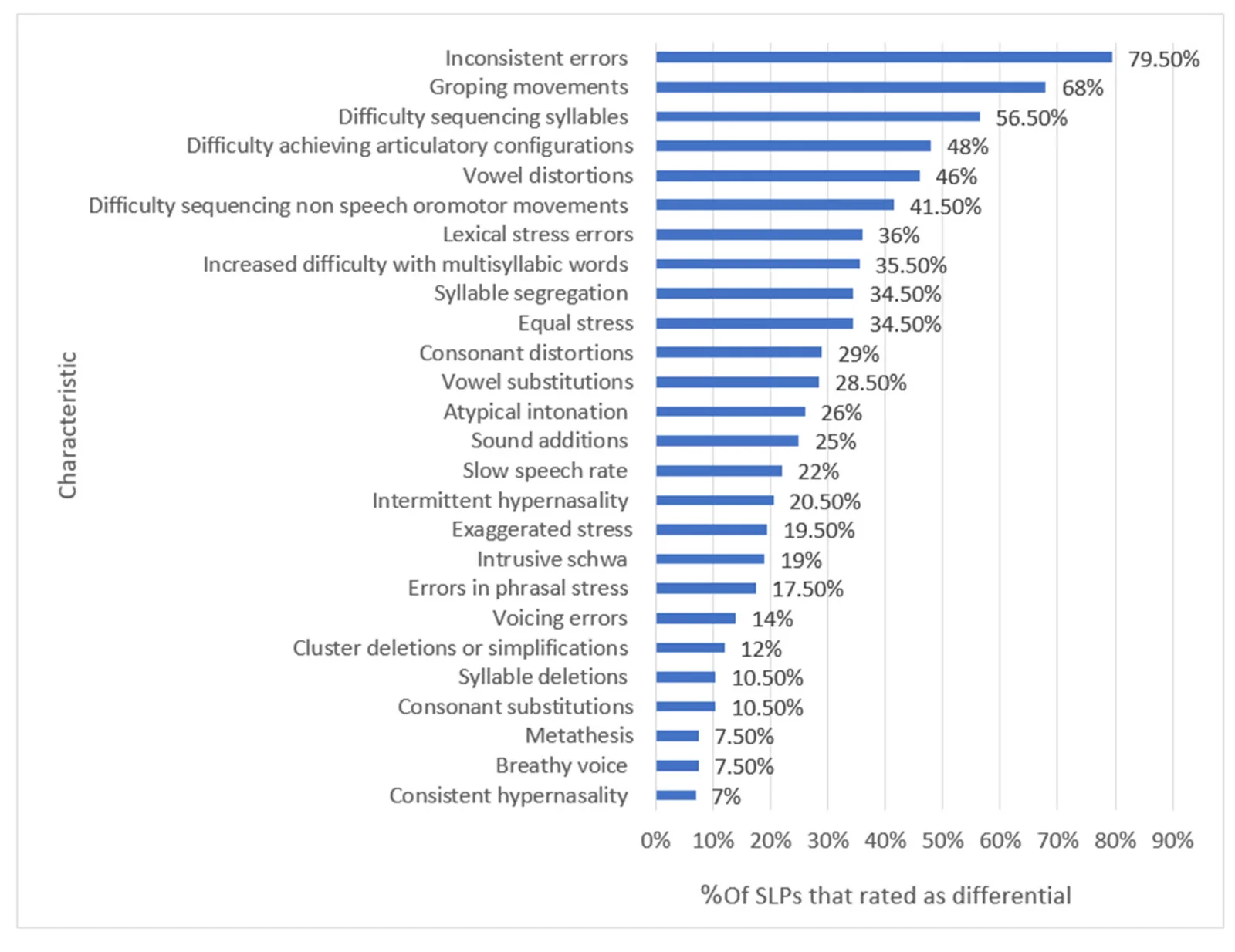
Percentage of SLPs rating each speech characteristic as diagnostically specific to CAS. Percentages reflect ratings of 6–7 on a 7-point specificity scale, where 1 = not specific at all and 7 = very specific. Breathy voice and consistent hypernasality were included as control characteristics and are not considered core features of CAS.

There was strong agreement among clinicians regarding the characteristics perceived as most diagnostically specific to CAS. Inconsistent errors received the highest level of endorsement, followed by groping movements and difficulty sequencing syllables. Nearly half of clinicians also identified difficulty achieving articulatory configurations and vowel distortions as diagnostically specific. Lexical stress errors, increased difficulty with multisyllabic words, syllable segregation, and equal stress patterns were endorsed by approximately one-third of clinicians, whereas consonant distortions and vowel substitutions received lower levels of endorsement. As expected, the control characteristics, breathy voice and consistent hypernasality, received the lowest diagnostic specificity ratings (Figure 5).

To examine whether clinicians’ endorsement of individual CAS characteristics differed according to years of professional experience, Mann–Whitney U tests compared SLPs with ≤10 years of clinical experience (60% of the sample) with those with >10 years of clinical experience (40%). The analyses were conducted separately for the percentage of SLPs rating each characteristic as common and the percentage rating each characteristic as diagnostic. A Bonferroni correction was applied to account for multiple comparisons, resulting in an adjusted significance level of *p* < 0.002. For characteristics rated as common, a nominal difference was found for syllable deletion (*p* = 0.04); however, this difference did not remain significant after Bonferroni correction. No significant differences were found for characteristics rated as diagnostic. Thus, after correction for multiple comparisons, no reliable differences between experience groups were observed, indicating that endorsement of individual CAS characteristics was generally consistent across levels of professional experience.

## 4. Discussion

The present study examined the common and diagnostic speech characteristics of CAS in Hebrew-speaking children through the perspectives of Israeli SLPs. Several key findings emerged. Increased difficulty with multisyllabic words, inconsistent errors, and difficulty sequencing syllables received the highest frequency ratings and were endorsed by the largest proportion of clinicians as common characteristics of CAS. In addition, inconsistent errors, groping movements, and difficulty sequencing syllables received the highest diagnostic specificity ratings, suggesting that clinicians view these characteristics as particularly informative for differential diagnosis. In contrast, prosodic features, including lexical stress errors and equal stress patterns, received ratings at or slightly below the midpoint of the scale and were endorsed by relatively few clinicians as common characteristics of CAS. Finally, breathy voice and consistent hypernasality, two control characteristics not typically associated with CAS, received the lowest ratings, supporting the validity of clinicians’ responses.

### 4.1. Perceived Frequency and Diagnostic Value of CAS Characteristics

The first important finding relates to the perception of frequent CAS characteristics among Israeli SLPs. In this study, Israeli SLPs identified several characteristics as highly frequent in Hebrew-speaking children with CAS. The five most observed features were increased difficulty with multisyllabic words, inconsistent errors, difficulty achieving articulatory configurations, cluster deletions or simplifications, and difficulty sequencing syllables.

These findings show strong alignment with the ASHA (2007) consensus criteria for CAS [1]. The frequent observation of inconsistent errors directly matches the first ASHA diagnostic criterion and is consistent with Tubul-Lavy’s (2012) study of Hebrew-speaking children, which showed that children with CAS demonstrate more variable repetitive productions compared to typically developing children [19]. The high frequency of difficulty with multisyllabic words reflects the recognition by ASHA that CAS features change in their relative frequencies of occurrence depending on task complexity.

This pattern is further consistent with cross-linguistic findings. For example, Shakibayi et al. (2019) found that Persian-speaking SLPs similarly identified inconsistent production, sequencing problems, and difficulties with multisyllabic words as primary features of CAS [12]. Malmenholt et al. (2017) found that Swedish clinicians reported parallel patterns, with inconsistent speech production, sequencing difficulties, and consonant cluster deletions ranking among the most characteristic features [10]. Using a different methodological approach, Chenausky et al. (2020) conducted a factor analysis of speech samples of American English-speaking children with CAS and found similar patterns, with inconsistency and coarticulatory difficulties among the key features identified [7].

The second main finding relates to diagnostic specificity, where Israeli SLPs identified five features as most indicative of CAS: inconsistent errors, groping movements, difficulty sequencing syllables, difficulty achieving articulatory configurations, and difficulty sequencing non-speech oromotor movements.

The identification of inconsistent errors and groping movements as the most diagnostically specific features is consistent with established theoretical frameworks of CAS. Inconsistent errors are thought to reflect the underlying impairment in speech motor planning and programming that characterizes CAS, resulting in difficulty producing stable and accurate speech movements across repeated attempts [1,2,9]. Groping movements, observed as visible trial-and-error articulatory searching behaviors, likewise reflect difficulties in planning and programming speech movements and are widely recognized as a hallmark clinical feature of CAS [1,2,9].

Similar patterns of diagnostic specificity have been reported internationally. Randazzo (2019) surveyed 165 speech–language pathologists, primarily in the United States, and found that inconsistent errors were rated as the most diagnostically important characteristic of CAS, followed by groping movements [15]. These findings closely correspond to the two highest-rated diagnostic features identified by Israeli SLPs in the present study. This pattern is further supported by Murray et al. (2021), whose systematic review identified inconsistent errors, disrupted coarticulatory transitions, and inappropriate prosody as the most consistently supported diagnostic features of CAS, while also highlighting the diagnostic value of tasks assessing syllable sequencing, such as sequential motion rates (e.g., /pʌtʌkʌ/) [9]. Taken together, these findings suggest that inconsistent errors, groping movements, and sequencing difficulties represent robust clinical indicators of CAS across languages.

One notable finding was the relatively low endorsement of prosodic characteristics in Hebrew-speaking children with CAS. Prosodic features received ratings at or slightly below the midpoint of the 7-point frequency scale, with fewer than 20% of clinicians identifying lexical stress errors (16.5%), equal stress patterns (16.5%), and errors in phrasal stress (17.5%) as common characteristics of CAS. Similarly, these features demonstrated relatively low diagnostic specificity: lexical stress errors (*M* = 4.44) and equal stress patterns (*M* = 4.42) received ratings only slightly above the midpoint of the specificity scale and were endorsed as diagnostically specific by approximately one-third of clinicians (36.0% and 34.5%, respectively). Together, these findings suggest that Israeli SLPs perceive prosodic features as neither highly prevalent nor particularly diagnostic of CAS in Hebrew-speaking children.

This pattern contrasts with reports from clinicians working with English-speaking children with CAS, among whom 49% identified lexical stress errors as a common characteristic of the disorder [15]. Among Swedish speech–language pathologists, lexical stress errors were endorsed as a common characteristic by 53% of respondents [10]. In contrast, speech–language pathologists from Estonia, Lithuania, and Finland did not consider prosodic features to be among the most diagnostically informative characteristics of CAS [14], a pattern that was similarly observed among Cantonese-speaking clinicians [23].

Taken together, these cross-linguistic findings suggest that clinicians in several non-English-speaking contexts may perceive prosodic features as less central to CAS diagnosis than clinicians working with English-speaking children with CAS. These observations raise important questions regarding the universal applicability of English-based diagnostic frameworks and highlight the need for language-specific investigations of CAS characteristics. Notably, the relatively high endorsement of lexical stress abnormalities in both English-speaking and Swedish contexts may reflect shared linguistic properties of these stress-based languages. However, additional cross-linguistic research is needed to determine which specific language features contribute to similarities and differences in the manifestation and diagnosis of CAS.

The relatively low endorsement of prosodic characteristics in Hebrew is consistent with recent evidence suggesting that Hebrew-speaking children with CAS demonstrate relatively accurate lexical stress production [18]. Tubi et al. (2024) proposed that these findings may reflect language-specific properties of Hebrew. Unlike English, Hebrew does not exhibit vowel reduction in unstressed syllables; vowel quality generally remains stable regardless of stress placement [17]. The absence of vowel reduction may facilitate more accurate lexical stress production and reduce the perceptual salience of stress errors in Hebrew-speaking children with CAS. Together, these findings suggest that the perceived frequency and diagnostic relevance of prosodic characteristics in CAS may vary across languages, potentially reflecting language-specific phonological and prosodic properties.

In addition to language-specific phonological properties, clinicians’ perceptions may also be shaped by their professional training and the diagnostic frameworks available to them. This interpretation is supported by Gomez et al. (2019), who reported considerable variation in the assessment and management of CAS among speech–language pathologists in Australia and New Zealand, highlighting the influence of clinical training and the need for evidence-based, language-specific guidance [24]. Because many CAS training materials and assessment guidelines have been developed based on English-speaking populations, Israeli SLPs may recognize lexical stress errors as an important diagnostic feature even if they encounter such errors relatively infrequently in Hebrew-speaking children. Together, these findings underscore the importance of developing and validating language-specific diagnostic frameworks while maintaining consistency with established international knowledge.

A noteworthy finding was the discrepancy between the perceived frequency and diagnostic value of lexical stress errors. Although only 16.5% of Israeli clinicians rated lexical stress errors as a common characteristic of CAS, 36.0% considered these errors diagnostically important when evaluating potential cases. This pattern suggests that clinicians may recognize lexical stress errors as a theoretically important marker of CAS, consistent with the ASHA (2007) consensus criteria, even though they observe these features relatively infrequently in Hebrew-speaking children [1].

### 4.2. Study Limitations

Several limitations should be considered when interpreting these findings. This cross-sectional survey relied on self-reported practices and perceptions rather than direct observation of clinical behavior, which may introduce response bias or discrepancies with actual practices. Importantly, the study examined clinicians’ perceptions of CAS characteristics rather than direct assessment of children’s speech patterns, and these perceptions may be influenced by theoretical knowledge derived predominantly from English-based literature rather than direct language-specific clinical evidence. In addition, participants’ professional qualifications and eligibility criteria were based on self-report and were not independently verified. Although respondents were screened according to the study’s inclusion criteria, it was not possible to confirm the accuracy of the information provided.

While an effort was made to distribute the questionnaire widely through multiple channels, the online format may have limited access for some clinicians. The sample may not be fully representative of all Israeli SLPs.

### 4.3. Clinical Implications and Future Research

This study provides the first comprehensive examination of CAS clinical practices and beliefs among SLPs. While Israeli clinicians identified several core characteristics of CAS that are consistent with the international literature, relatively low endorsement of prosodic features, particularly lexical stress errors, suggests potential cross-linguistic variation in the manifestation and perception of CAS. These findings raise important questions regarding the universal applicability of English-based diagnostic frameworks and underscore the need for further language-specific investigations of CAS characteristics in Hebrew-speaking children.

Future research should focus on directly examining the speech characteristics of Hebrew-speaking children with CAS to better identify language-specific diagnostic markers. Such work may help clarify whether the clinical features currently emphasized in English-based frameworks are equally relevant in Hebrew. An additional priority is the development and validation of Hebrew-appropriate assessment tools, including adaptation of existing measures and creation of new clinically sensitive instruments. Expanding research in this area may also support improved clinician training and contribute to greater diagnostic confidence and consistency among SLPs.

## Figures and Tables

**Table 1:** Summary of previous cross-linguistic studies examining speech–language pathologists’ perceptions of speech characteristics of childhood apraxia.

Study	Language	Participants	Most Common/Perceived Diagnostic Characteristics	Prosodic Findings
Randazzo (2019) [15]	English	Speech–language pathologists	Inconsistent errors, groping movements, and lexical stress errors were among the characteristics most frequently perceived as common and diagnostically important.	Lexical stress errors were considered an important diagnostic characteristic (49% of clinicians endorsed them as common).
Malmenholt et al. (2017) [10]	Swedish	Speech–language pathologists	Inconsistent errors and sequencing difficulties were highly endorsed.	Lexical stress errors were considered relatively common (53%).
Shakibayi et al. (2019) [12]	Persian	Speech–language pathologists	Inconsistent errors, multisyllabic word difficulties, and sequencing deficits received the highest ratings.	Prosodic characteristics received lower ratings than word-level characteristics.
Wong et al. (2020) [13]	Cantonese	Expert speech–language pathologists (Modified Delphi survey)	Inconsistency and sequencing difficulties were identified as core characteristics of CAS.	Prosodic characteristics were considered less diagnostically informative than in English.
Lahtein-Kürsa et al. (2025) [14]	Estonian, Finnish, Lithuanian	Speech–language pathologists	Inconsistent errors and sequencing difficulties were highly endorsed across all three languages.	Lexical stress errors and equal stress patterns were not considered highly diagnostic.

**Table 2:** Demographic information of the participants.

Demographic Descriptors	Number of Respondents (%)
Gender	
Female	197 (98.5%)
Male	3 (1.5%)
Age	
20–29	35 (17.5%)
30–39	107 (53.5%)
40–49	44 (22.0%)
50–59	10 (5.0%)
60–69	3 (1.5%)
70 and over	1 (0.5%)
Native Language	
Hebrew	191 (95.5%)
English	5 (2.5%)
Russian	1 (0.5%)
French	1 (0.5%)
Hebrew + English	2 (1.0%)
Education	
Bachelor’s degree	117 (58.5%)
Master’s degree	78 (39.0%)
PhD or higher	5 (2.5%)
Years of experience	
Under 5 years	44 (22.0%)
5–10 years	76 (38.0%)
11–15 years	37 (18.5%)
16–20 years	17 (8.5%)
Over 20 years	26 (13.0%)

Note: Hebrew + English indicates participants reporting both languages as native.

**Table 3:** Work settings.

Types of Work Settings (Multiple Responses Possible)	
Hospital	11 (5.5%)
Public speech and language clinic	22 (11.0%)
Private speech and language clinic	82 (41.0%)
Child habilitation services	3 (1.5%)
Adult habilitation services	2 (1.0%)
Special education settings	21 (10.5%)
Special pre- and primary schools for children with speech and language disorders	79 (39.5%)
Child development center	69 (34.5%)
Independent private practices	38 (19.0%)
Hearing clinic	4 (2.0%)

Note: N = 200 for the number of work settings. Total responses in types of work settings exceed N because respondents could select multiple answers.

## Data Availability

The data presented in this study are available from the corresponding author upon reasonable request. The data are not publicly available due to ethical and privacy considerations related to participant confidentiality.

## Appendix A. Clinician Questionnaire

Perceived Frequency and Diagnostic Value of Childhood Apraxia of Speech (CAS) Characteristics

Instructions

Clinicians were asked to rate a series of speech characteristics associated with childhood apraxia of speech (CAS). For each characteristic, participants provided two ratings using a 7-point Likert scale:

1. Frequency Rating: *How common is this characteristic in children with CAS?*
  (1 = Not common at all; 7 = Very common)
2. Diagnostic Rating: *How indicative is this characteristic of CAS?*
  (1 = Not indicative at all; 7 = Highly indicative)

**Table A1:** Characteristics Rated.

#	Characteristic
1	Vowel distortions (imprecise production of the intended vowel)
2	Vowel substitutions (substituting one vowel for another; e.g., *bear* → *bar*)
3	Consonant substitutions (substituting one consonant for another; e.g., *cat* → *tat*)
4	Difficulty achieving articulatory configurations and movement transitions (e.g., lip rounding or transitions between sounds and syllables)
5	Equal stress across syllables (i.e., Using equal emphasis on all syllables, such as pronouncing “banana” as “BUH-NAN-UH” instead of “buh-NAN-uh”)
6	Lexical stress errors (incorrect word-level stress patterns, e.g., pronouncing “banana” as “BUH-nan-uh” instead of “buh-NAN-uh”)
7	Exaggerated stress (an exaggerated emphasis on the stressed syllable)
8	Consonant distortions (imprecise production of the intended consonant)
9	Syllable segregation (pauses or gaps between syllables)
10	Groping movements of the jaw, lips, or tongue during speech attempts
11	Intrusive schwa (insertion of an additional vowel sound; e.g., *play* → *pe-lay*)
12	Voicing errors (e.g., *down* → *town*)
13	Slow speech rate
14	Increased difficulty with multisyllabic words
15	Errors in phrasal stress (e.g., excessive/equal stress throughout a sentence)
16	Atypical intonation (e.g., talking in a monotonous intonation)
17	Breathy voice quality
18	Consistent hypernasality
19	Syllable deletions (e.g., *banana* → *nana*)
20	Sound additions
21	Inconsistent errors across repeated productions of the same word
22	Intermittent hypernasality
23	Difficulty sequencing non-speech oral movements
24	Difficulty sequencing syllables (e.g., during diadochokinetic tasks such as *pa-ta-ka*)
25	Cluster deletions or simplifications (e.g., *blue* → *boo*)
26	Metathesis (reversal of sounds within a word; e.g., *potato* → *topato*)

Note: Each characteristic was rated separately for (a) perceived frequency in CAS and (b) diagnostic value for CAS using a 7-point Likert scale. This format was adapted from the questionnaire administered to participants.
